# Genetic epidemiology of Woodhouse-Sakati Syndrome in the Greater Middle East region and beyond: a systematic review

**DOI:** 10.1186/s13023-023-02614-8

**Published:** 2023-01-31

**Authors:** Amira Kohil, Atiyeh M. Abdallah, Khalid Hussain, Mashael Al-Shafai

**Affiliations:** 1 Department of Biomedical Sciences, College of Health Sciences, QU Health, Qatar University, 2713 Doha, Qatar; 2 Department of Pediatrics, Division of Endocrinology, Sidra Medicine, Doha, Qatar

**Keywords:** Woodhouse-Sakati, Variants, *DCAF17*, Arabs, Middle East, Consanguinity

## Abstract

**Background:**

Woodhouse-Sakati syndrome (WSS) is a rare, autosomal recessive genetic disorder with variable clinical manifestations mainly affecting the endocrine and nervous systems. The aim of this study was to systematically review the genetic basis of WSS and report the genetic variants and clinical phenotypes associated with the disease.

**Methods:**

PubMed, Science Direct, Scopus, and Web of Science databases were searched from the time of inception until June 2022. Broad search terms were used to capture the literature describing all genetic variants associated with WSS. The search keywords used are “Woodhouse Sakati” along with the term “mutation” OR “gene” OR “variant” OR “polymorphism”.

**Results:**

Twenty-five eligible studies were included in this study. One hundred and eighty-five patients in 97 families from 12 different countries were diagnosed with WSS. In patients from the Greater Middle East (GME) region, consanguineous marriages were common (67%). Thirteen different *DCAF17* variants were associated with WSS development (including 8 identified in the GME region). The most frequent variant was a frameshift deletion variant (c.436delC, p.Ala147Hisfs*9) unique to Arabs that was reported in 11 cases from Tunisia, Kuwait, Qatar, Bahrain, and Saudi Arabia. There were no clear genotype–phenotype correlations for the different variants.

**Conclusions:**

This systematic review highlights the molecular basis and clinical manifestations of WSS globally, including the GME region, where the disease is prevalent due to consanguinity. Additional studies are now needed to understand the genotype–phenotype correlation for different *DCAF17* variants and their impact on the phenotypic heterogeneity observed in WSS patients.

**Supplementary Information:**

The online version contains supplementary material available at 10.1186/s13023-023-02614-8.

## Background

Woodhouse-Sakati syndrome (WSS), first described in a consanguineous Saudi Arabian family in 1983 [1], is a rare, autosomal recessive genetic disorder [2]. WSS is characterized by a variety of predominantly endocrine and nervous system abnormalities including hypogonadism, diabetes mellitus (DM; in 95% of patients), hypothyroidism, low insulin-like growth factor (IGF-1) levels, deafness, alopecia, and electrocardiographic abnormalities [3, 4]. The prevalence of WSS is estimated to be < 1/1,000,000 of the population [2]. There is no clear age of onset for the disorder, but the different clinical manifestations can present at different times; for example, hypogonadism is often detected around the time of puberty (12–14 years of age); DM and hypothyroidism during adolescence up to the age of 25 years of age; and neurological manifestations between nine and 17 years of age [2].

Therefore, the clinical features of WSS are heterogeneous. On clinical examination, WSS patients characteristically have a flat occiput, triangular face, high forehead, frontal bossing, mild hypertelorism, short and sparse eyebrows, down-slanting palpebral fissures, a prominent nasal root, dental malocclusion, and a high-arched palate [4]. Other clinical features include progressive childhood-onset alopecia (leading to alopecia totalis) and the neurologic findings of progressive extrapyramidal movements (dystonic spasms with dystonic posturing with dysarthria and dysphagia), sensorineural hearing loss, and intellectual disability [5]. Brain MRI performed on some of these patients has revealed diffuse white matter disease and sometimes increased iron accumulation in the basal ganglia [6]. Regardless of the specific features seen in individuals, patients generally suffer severe morbidity and a high risk of early death, and there is currently no effective treatment for the condition.

WSS is caused by the inheritance of mutations in *DCAF17* (formerly known as *C2orf37*), and overexpression of nucleolar DCAF17 protein is associated with the WSS phenotype both clinically and in animal studies [7]. The two transcripts of *DCAF17* have unknown function but the encoded proteins localize to the nucleolus, where they are involved in cell cycle regulation. It has been suggested that mutant *DCAF17* leads to defective ribosome biogenesis, resulting in cell cycle abnormalities and cellular aging [8]. WSS is also associated with some loss of function mutations that reduce splicing efficiency and truncate the protein. However, there is still no clear genotype–phenotype correlation in WSS, partly due to the marked phenotypic variability and partly because the disease is so rare.

The diagnosis of WSS can be difficult due to this clinical heterogeneity and spectrum of common symptoms and signs, so molecular genetics is important to secure the diagnosis and for patient management. Moreover, once the pathogenic variant is identified in a family member, further genetic testing and counseling can be conducted to determine the individual risk of passing on/having the condition through carrier testing, prenatal testing, and pre-implementation genetic diagnosis [9]. Here we systematically reviewed the genetics and molecular basis and biology of WSS to shed further light on genotype–phenotype correlations observed globally.

## Results

### Search outcome

**Fig. 1:**
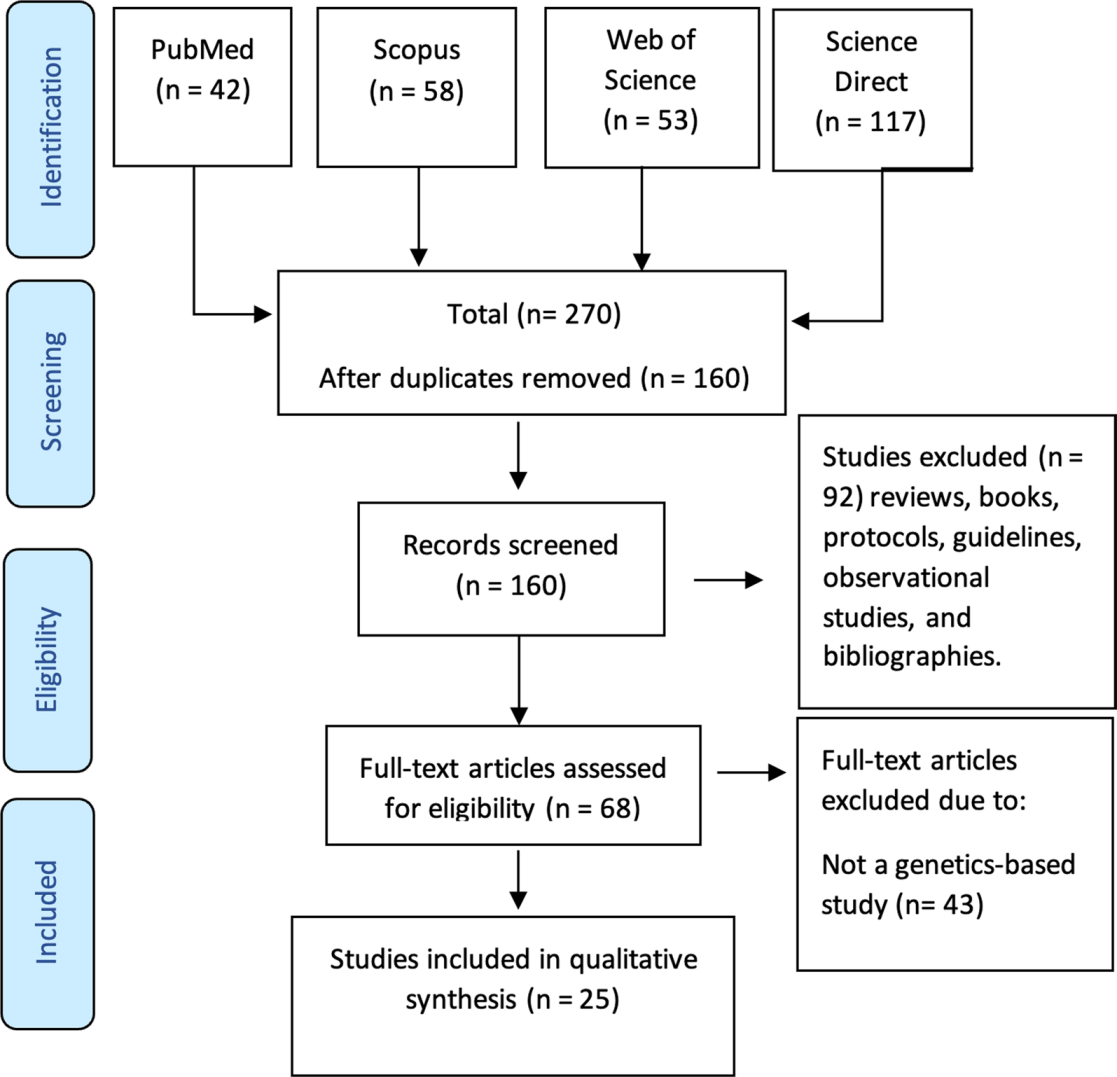
PRISMA flow chart detailing the search strategy and study selection process

The PRISMA flow chart is shown in Fig. 1. Our search strategy yielded 270 studies, of which 160 remained after removal of duplicates. The remaining articles were subjected to primary screening by title, abstract, and PICOS assessment for eligibility according to the inclusion and exclusion criteria (Table 1). Of the screened articles, 68 were eligible for full assessment. Of these, 44 articles were not genetic studies. Therefore, 25 articles were eligible for inclusion in this review.

**Table 1:** The inclusion and exclusion criteria according to the PICOS statement

	Included	Excluded
Population	All global populations	Irrelevant
Interventions	None	None
Comparators	Patient with WSS at presentation assessment	Articles with no genetic analysis
Study design	Prospective, retrospective cohort, case reports, and research articles	Reviews, books, protocols, guidelines, and animal studies
Primary outcome	Genetic variants reported in WSS patients	Irrelevant
Secondary outcomes	Clinical phenotype variability in WSS patients	Irrelevant

*PICO* population, intervention, comparator, outcomes, and study design, *WSS* Woodhouse-sakati syndrome

### Quality of the eligible studies

**Fig. 2:**
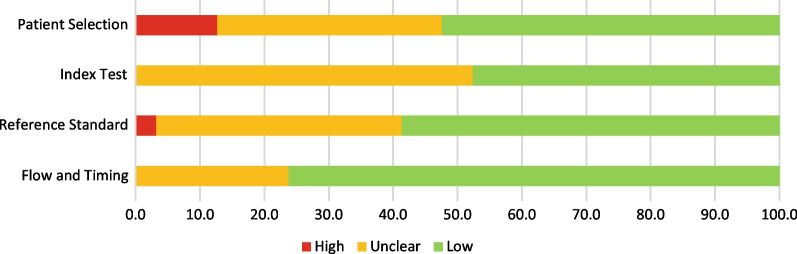
Risk of bias assessment using the QUADAS-2 tool

Risk of bias assessments in the four domains of the QUADAS-2 tool are shown in Fig. 2. The patient selection domain had the highest risk of bias, with a high risk of bias in 37.5% of included articles. This was expected, as we included case reports in which participants were preselected. 52.4% of studies were rated as having a low risk of bias, as a full description of the diagnostic threshold and the standardized procedure for the test was given. There was mainly a low risk of bias in the remaining three domains.

### Reported genetic variants

In the 25 included articles, WSS-associated mutations were reported in 185 patients belonging to 97 families. In most cases, genetic testing was performed by a mixture of Sanger and next-generation sequencing approaches. Consanguinity was observed in 67% (n = 65) of families, while only three families were from non-consanguineous marriages. In 29 families (29.8%), consanguinity was not specified.

Thirteen WSS-causing variants were described in the *DCAF17* gene. These were reported mainly in the Greater Middle East (GME) countries (Tunisia, Kuwait, Qatar, Bahrain, Saudi Arabia, Pakistan, Iran, Turkey), where consanguinity is common [10], but there were cases in four other countries (Portugal, China, Italy and India) (Table 2). The most frequently identified variant was a deletion mutation (rs797045038, c.436delC p.Ala147Hisfs*9) accounting for 11 cases reported in five countries (Tunisia, Kuwait, Qatar, Bahrain, and Saudi Arabia).

**Table 2:** The disease-causing variants in *DCAF17* reported in the literature in association with WSS

	Rs ID (gnomAD prevalence)	Nucleotide change (protein change)	Population	Study type	Sample description	Number of reported patients with the variants	Gender (number of patients)	Clin-Var	References
1	rs797045038	c.436delC p.Ala147Hisfs*9	Tunisia	Case report	A consanguineous family of six children	2 siblings	Female (2)	P	[11]
			Kuwait	Case series	A consanguineous family of seven children	2 siblings	Female (2)		[12]
			Qatar	Case series	A consanguineous family of nine children	1 patient	Female (1)		
			Bahrain	Case series	Consanguineous family	2 siblings	Female (1) Male (1)		
			Kuwait	Case report	A consanguineous family of ten children	3 siblings	Female (2) Male (1)		[13]
			Qatar	Clinical reports	Two consanguineous families (18 subjects)	Family 1: 3 siblings Family 2: 4 siblings	Female (3) Male (4)		[14]
			Qatar	Case report	A consanguineous family with 7 children	2 siblings	Female (1) Male (1)		[15]
			Qatar	Case report	One family	3 siblings	-		[16]
			Qatar	Research article	58 subjects from 32 consanguineous families	58 patients	Female (34) Male (24)		[17]
			Saudi Arabia	Research article	184 subjects	18 patients	-		[18]
			Saudi Arabia	Case report	A consanguineous family (3 subjects)	3 patients	Female (3)		[19]
			Saudi Arabia	Case series	38 subjects (from 17 families)	38 patients	Female (18) Male (20)		[20]
			Saudi Arabia	Research article	13 patients (from 9 families)	13 patients	Male (5) Female (8)		[21]
			Unspecified (Middle East)	Case report	A consanguineous family with four children	2 siblings	Female (1)		[22]
2	(0.0001)	c.1A > G	Pakistan	Research article	Four generation consanguineous family	2 siblings	Male (2)	NA	[23]
3	rs879253799 (0.000004)	c.270delA (p.Lys90Asnfs*8)	Pakistan	Short report	Four generation consanguineous family	4 siblings	Female (2) Male (2)	P	[24]
4	–	c.321 + 1G > A	Pakistan	Research article	Four generation consanguineous family	6 siblings	Female (3) Male (3)	NA	[25]
5	–	c.1091 + 1G > A	Turkey	Case report	A consanguineous family with six children	1 patient	Female (1)	NA	[26]
			Iran	Case report	–	1 patient	Male (1)	NA	[27]
6	–	c.270dup (p.Cys91Metfs*28)	Turkey	Clinical report	A consanguineous family	1 patient	Female (1)	P	[28]
7	–	c.127-3delTAGinsAA	Turkey	Short report	7 patients from four consanguineous families	1 patient	Male (1)	P	[29]
8	–	c.1423‐1_1425delGACA	Pakistan	Research article	Four generation consanguineous family	1 patient	Male (2) Female (3)	NA	[30]
9	–	c.1091 + 2 T > C	Portugal	Case report	One consanguineous family with a single child	1 patient	Female (1)	P	[31]
10	rs778488574 (0.000011)	c.1488_1489delAG	China	Case report	One non-consanguineous family with four children	2 patients	Male (1) Female (1)	NA	[3]
11	–	c.1111delA	China	Case report	One consanguineous family with three children	2 patients	Male (1) Female (1)		[32]
12	–	c.906 G > A	Italy	Short report	One family	3 patients	Male (1) Female (2)		[33]
12		c.1238delA	India	Case report	One non-consanguineous family with four children	3 patients	Male (1) Female (2)		[34]
13	–	c.459- 7_499del	India	Case report	One non-consanguineous family with four children	3 patients	Male (1) Female (2)		[34]

### Clinical phenotypes

Additional file 1: Table S1 shows the reported phenotypes in the 185 patients from the 25 included articles. Endocrine manifestations of hypogonadism and DM were seen in all patients, as was ectodermal involvement. The majority of patients showed neurological involvement. Some patients presented with high degree of intellectual disability. Other less common phenotypes included sensorineural hearing loss, dystonia, dysarthria, and other symptoms. Some studies reported the radiological findings from brain MRI, namely iron deposition within the substantia nigra and globus pallidus and subcortical white matter changes.

### Genotype–phenotype correlation

**Fig. 3:**
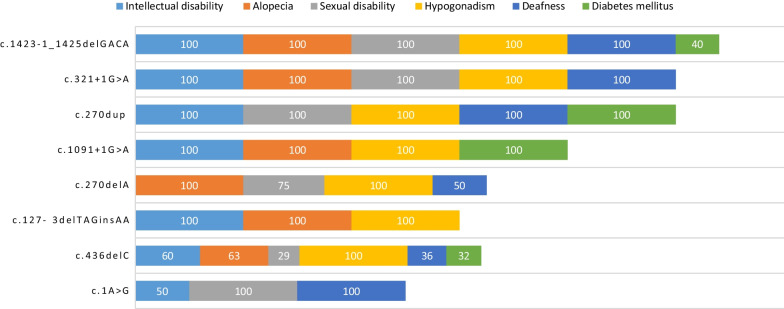
Distribution of the six most common clinical features according to *DECAF17* variant

Table 3 shows the distribution of genetic variants according to disease phenotypes. No formal statistical analysis of the distribution of genetic variants with clinical manifestations was conducted, as the sample numbers were too low. Figure 3 shows the distribution of the six most common clinical features according to different *DECAF17* variants. Intellectual disability was reported for all genetic variants except c.270delA. Alopecia was reported for all variants except c.270dup and c.1A > G. In addition, hypogonadism was reported in all genetic variants reported in this study. Therefore, there was no clear genotype–phenotype correlation identified in these patients.

**Table 3:** The distribution of clinical features according to genetic variants of *DCAF17* reported in the literature

Genetic variant	Clinical manifestations								
	Endocrine				Ectodermal	Neurological		Ocular	
	Hypogonadism	Diabetes mellitus	Hypothyr-oidism	Alopecia	Deafness	Intellectual disability	Extrapyramidal features	Keratoc-onus	Myopia
c.1423‐1_1425delGACA	5/5 (100%)	2/5 (40%)	–	5/5 (100%)	5/5 (100%)	5/5 (100%)	1/5 (20%)	–	–
c.270dup	1/1 (100%)	1/1 (100%)	1/1 (100%)	–	1/1 (100%)	1/1 (100%)	–	–	–
c.1091 + 1G > A	1/1 (100%)	1/1 (100%)	–	1/1 (100%)	–	1/1 (100%)	–	–	–
c.270delA	4/4 (100%)	–	–	4/4 (100%)	2/4 (50%)	–	–	–	–
c.127-3delTAGinsAA	1/1 (100%)	–	1/1 (100%)	1/1 (100%)	–	1/1 (100%)	1/1 (100%)	–	–
c.321 + 1G > A	6/6 (100%)	–	–	6/6 (100%)	6/6 (100%)	6/6 (100%)	3/6 (50%)	–	–
c.436delC	133/133 (100%)	38/133 (28%)	27/133 (20%)	76/133 (57%)	49/133 (37%)	72/133 (54%)	46/133 (34%)	54/133 (41%)	56/133 (42%)
c.1A > G	2/2 (100%)	–	–	–	2/2 (100%)	1/2 (50%)	–	–	–
c.1091 + 2 T > C	1/1 (100%)	1/1 (100%)	1/1 (100%)	1/1 (100%)	1/1 (100%)	1/1 (100%)	1/1 (100%)	–	–
c.1488_1489delAG	2/2 (100%)	2/2 (100%)	2/2 (100%)	2/2 (100%)	–	2/2 (100%)	–	–	–
c.1111delA	2/2 (100%)	2/2 (100%)	2/2 (100%)	2/2 (100%)	–	2/2 (100%)	–	–	–
c.906 G > A	3/3 (100%)	2/3 (66%)	–	3/3 (100%)	2/3 (66%)	3/3 (100%)	2/3 (66%)	–	–
c.1238delA	3/3 (100%)	3/3 (100%)	–	3/3 (100%)	3/3 (100%)	3/3 (100%)	–	–	–
c.459- 7_499del	3/3 (100%)	3/3 (100%)	–	3/3 (100%)	3/3 (100%)	3/3 (100%)	–	–	–

## Discussion

To our best knowledge, this is the first comprehensive systematic review of the variants associated with WSS. We retrieved twenty-five published papers representing 185 patients in 97 families with WSS-associated variants from 12 countries globally. This included 171 patients (93%) from eight GME countries, where consanguinity is common. Marriages were consanguineous in the majority (67%) of the WSS families identified, which was expected given that WSS is an autosomal recessive condition and consanguinity increases the likelihood of inheriting two copies of a deleterious allele from a common ancestor (autozygosity). This explains why most cases of WSS are reported in the GME region.

Most of the available data on WSS cases came from case reports and series. As a rare genetic disorder, case reports and case series are a helpful tool, since they provide knowledge that is informative to systematic reviews, especially when data are scarce from randomized controlled trails and observational studies [35].

A study conducted by Abouelhoda et al., identified 259 homozygous mutations in a cohort of 7,000 unrelated patients from Saudi Arabia. They showed that *DCAF17* combined carrier frequency is 0.003 ( PMID: 27,124,789) [36]. In our study, we found that the most common variant identified was the c.436delC variant on exon 4 of *DCAF17*. This variant was found mainly in Arabs and was reported in five Arab countries (Tunisia, Kuwait, Qatar, Bahrain, and Saudi Arabia), mainly in consanguineous families. This variant is in the open reading frame (ORF) of *DCAF17* and is predicted to cause a frameshift mutation leading to the production of a prematurely truncated protein (p.Ala147Hisfs*9). The resulting protein is predicted to have 520 amino acids with no significant homology with other proteins [18]. The homozygous form of the c.436delC variant was reported in almost one-half of the reported cases in the Arab population, specifically in the Gulf region, so we consider this variant to be a founder variant in this population [12]. Abouelhoda et al. identified *DCAF17* as a founder mutation in Arabic regions [36]. Another exonic variant associated with WSS development was the c.270delA (p.Lys90Asnfs8*) variant on exon 3 of *DCAF17*, which was identified in a Pakistani family (Table 1). This is a single base pair frameshift deletion affecting both gene transcripts to produce a truncated protein of 96 amino acids [24]. In addition, a deletion mutation (c.1488_1489delAG) and a nonsense mutation (c.1111delA) were reported in Chinese WSS patients, leading to the formation of a premature stop codon and thus predicted to form a truncated protein [3, 32]. Another exonic variant was also reported in India (c.1238delA), which results in a frameshift variant in exon 12, which is predicted to cause premature protein truncation [34]. The truncated DCAF17 protein resulting from these variants lacks the domains and motifs needed for interactions with the DDB1‐CUL4 ubiquitin ligase complex. The inefficient interaction between the truncated DCAF17 protein and other proteins could affect several functions within the nucleoli contributing to WSS [37–39].

A loss-of-function variant was also detected in the first start codon of *DCAF17* in a Pakistani family suffering from WSS. This variant affects the translation initiation codon of *DCAF17*, which is predicted to inhibit the translation of its protein [23]. Six different splice site variants (c.127‐3delTAGinsAA, c.1091 + 1G > A, c.321 + 1G > A, c.1423‐1_1425delGACA, c.1091 + 2 T > C, and c.459- 7_499del) leading to exon skipping were found in different populations including patients from Pakistan, Turkey, Iran, Portugal, and India. It is well known that splice site variants contribute to the pathogenesis of different human genetic disorders, as around 15% of such variants affect pre-mRNA splicing [40]. Splice site variants cause inefficient splicing that could lead to exon skipping, intron retention, cryptic splice site activation, and the production of pseudo-exons within an intron [41].

*DCAF17* is known to produce two main transcripts (alpha and beta isoforms) whose protein products localize to the nucleolus to exert as yet unknown functions in the human cell cycle. Both *DCAF17* transcripts are ubiquitously expressed in adult human tissues, so the predominance of ectodermal, endocrine, and neurological involvement in WSS is not fully understood [12]. Alazami and colleagues suggested that WSS is caused by a nucleolar defect resulting from aberrant *DCAF17* gene function that results in lymphoblast hypersensitivity to transcriptional blockade [18]. This study also observed that, in WSS, *DCAF17* gene expression is high in the brain, skin, and liver, further confirming the pleiotropic nature of the disorder [18]. Similar to WSS, variants in other nucleolar proteins have been associated with a multisystem disorder of multiple endocrinopathies, alopecia, and hypogonadism [42]. Therefore, the nucleolus is likely to play an important role in the pathophysiology of these disorders due to its important role in several physiological processes [43]. All known variants reported in *DCAF17* and found to be associated with WSS development possessed a full mutational spectrum covering the entire coding region. However, the nature and severity of phenotypes observed in WSS patients were not correlated with the expected length of the produced protein. Therefore, an intact full-length protein is probably needed for proper functionality, with the resulting protein sensitive to disruption of even a few amino acids [29]. Furthermore, since the length of the truncated protein in WSS patients does not seem to explain the clinical presentation, nonsense‐mediated decay (NMD) of mRNA and inefficient interactions of the truncated protein with other partners could explain the range of phenotypes observed [24]. The genotype–phenotype correlation in WSS still needs to be established.

This study has several limitations, mainly a lack of published peer-reviewed genetic studies related to WSS. Therefore, conducting a meta-analyses to ascertain the concordance of variants between patients was not possible.

## Conclusions

In conclusion, this systematic review, reports 13 variants associated with WSS development and its pathogenesis. We consider this systematic review to be the first to discuss the variants associated with WSS globally, including in the GME region, where the condition seems to be most prevalent due to consanguinity. The genotype–phenotype correlation in WSS still needs to be established, as most reported cases displayed marked phenotypic variability. This lack of correlation indicates the presence of unknown modifier genes or epigenetic processes that modify the disease course. Further studies are needed to understand the mechanisms underlying the *DCAF17* variants and their implication for the phenotypic heterogeneity observed in WSS patients. Ultimately, examining the genetic and molecular basis of WSS will improve our understanding of the disorder, nucleolar function, and improve the management of patients and their families.

## Methods

### Search strategy

The PubMed, Science Direct, Web of Science, and Scopus databases were searched from their dates of inception until June 2022. An extensive search of WSS studies globally was conducted using keywords constructed to include our primary or secondary outcomes. The primary outcome of this study was to report the genetic variants associated with WSS globally. The secondary outcome was to report the clinical phenotype variability observed in WSS patients (Table 1). Therefore, the search keywords included: “Woodhouse Sakati” along with the term “mutation” OR “gene” OR “variant” OR “polymorphism”. Initial screening (based on the abstract and title of the study) was performed on all the retrieved articles. Articles that met the inclusion criteria were fully evaluated and were included in this review.

### Study selection

Retrieved records were assessed by two researchers, and the senior author resolved any disagreement by consensus. The total number of hits from each database was recorded. Research papers that met the following inclusion criteria were chosen for full assessment: (1) articles from peer-reviewed journals; and (2) the articles discussed genetic variants associated with WSS. Research papers were excluded if at least one of the following exclusion criteria was met: (1) articles lacking genetic information on WSS; and (2) the published material was a review, book, protocol, or guideline or animal studies (Table 1). After removal of duplicates, the titles and abstracts of the remaining articles were assessed. All records not matching our inclusion criteria were removed. Secondary selection included assessment of the entire full text to collect relevant records. Two researchers performed the assessment and a senior researcher helped to solve any disagreement. Figure 1 is a PRISMA flow chart showing the screening and selection process.

### Quality control assessment and data extraction

Two researchers evaluated the quality of eligible articles for risk of bias using the Quality Assessment of Diagnostic Accuracy Studies-2 (QUADAS-2) tool [44]. This tool assigns a risk rating to four sections: patient selection, index test, reference standard, and flow and timing. Each section was rated as “low risk”, “high risk”, or “unclear risk”. In case of discrepancy, a senior opinion was sought.

Data items were collected from the tables and text of eligible articles and collated in a Microsoft Excel spreadsheet. Two researchers reviewed the collected data for accuracy. The data variables extracted included the name and function of the gene, sample size, genetic test used, number of patients tested and proportion with the causative variant, zygosity, consanguinity, gender, and phenotypic information. All captured variants were checked in PubMed (SNP), ClinVar (https://www.ncbi.nlm.nih.gov/clinvar/), Exome Variant Server (EVS; http://evs.gs.washington.edu/EVS/), Human Gene Mutation Database (HGMD; http://www.hgmd.cf.ac.uk/ac/index.php), and Google Scholar to obtain further insights on the variants identified.

## Supplementary Information

**Additional file 1**. The clinical features associated with the genetic variants in *DCAF17* reported in the literature.
