# Supplementary material for: Genetic epidemiology of Woodhouse-Sakati Syndrome in the Greater Middle East region and beyond: a systematic review
Source: Orphanet J Rare Dis. 2023 Jan 31;18:22. doi: 10.1186/s13023-023-02614-8 (PMC9887781; doi:10.1186/s13023-023-02614-8)
Supplement: Supplementary file 1 — Additional file 1. The clinical features associated with the genetic variants in DCAF17 reported in the literature. [file 13023_2023_2614_MOESM1_ESM.docx]

**Supplementary Table 1.** The clinical features associated with the genetic variants in DCAF17 reported in the literature.

| **#** | **Genetic variant** | **Ref.** | **Clinical phenotype** |
| --- | --- | --- | --- |
| 1 | c.436delC | [1] | Progressive appearance of abnormal posturing and twisting movements, absence of secondary sexual characteristics, amenorrhea, diabetes mellitus, generalized dystonia, intellectual disability , edentulism, alopecia |
|  |  | [2] | Alopecia, intellectual disability, deafness, hypoplastic uterus, hypothyroidism, hyperlipidemia, diabetes mellitus along with end-stage renal disease, progressive gait deterioration, epilepsy, and T-wave abnormalities on ECG |
|  |  |  | Patient 1: Hypotrichosis, focal choreoathetoid, dystonic movements, progressive gait abnormalities, dysarthria, moderate intellectual impairment, cerebral palsy with extrapyramidal signs, delayed puberty, growth retardation, hypothyroidism, mild hypotrichosis, uncorrected visual acuity, and mild scoliosis  Patient 2: Frontotemporal hypotrichosis, intellectual disability, hypoplastic uterus, absence of both ovaries, uncorrected visual acuity and myopic fundus in both eyes  Patient 3: Frontotemporal hypotrichosis, intellectual disability, extrapyramidal abnormalities, delayed puberty, growth retardation, uncorrected visual acuity, and hypothyroidism |
|  |  |  | Family 1:  Patient 1: Delayed puberty, myopia and moderate sensorineural hearing loss, partial frontal alopecia, and borderline intellectual disability  Patient 2: Delayed puberty, primary amenorrhea, frontal alopecia, bilateral keratoconus, bilateral mild sensorineural deafness, borderline intellectual disability, and a hypoplastic uterus  Patient 3: Delayed puberty, primary amenorrhea, frontal alopecia, myopia, mild sensorineural hearing loss, borderline intellectual disability, and a hypoplastic uterus  Family 2:  Patient 4: Delayed puberty, primary amenorrhea, partial alopecia, borderline intellectual disability, and mild sensorineural deafness  Patient 5: Delayed puberty, hyperactivity, mild intellectual disability, and partial alopecia  Patient 6: Hyperactivity, mild intellectual disability, partial alopecia, and white matter hypomyelination  Patient 7: Hyperactivity, mild intellectual disability, and partial alopecia |
|  |  | [3] | Patient 1: Hypogonadotropic hypogonadism, lack of sexual development, alopecia, delayed primary and secondary tooth eruption, hyperopia, myopia with astigmatism, and microcephaly  Patient 2: Primary amenorrhea, hypoplastic ovaries and uterus |
|  |  | [4] | Hypogonadism, alopecia, diabetes mellitus, and different degrees of intellectual disability ranging from mild to severe |
|  |  | [5] | Hypogonadism, diabetes mellitus, alopecia, hypothyroidism, keratoconus, and deafness |
|  |  | [6] | Hypogonadism, alopecia, diabetes mellitus, intellectual disability, and extrapyramidal features |
|  |  | [7] | Patient 1: Mild extrapyramidal symptoms, primary amenorrhea, gonadal dysfunction, diabetes mellitus, alopecia, hypothyroidism, and lack of sexual development  Patient 2 and 3: Primary amenorrhea, alopecia, diabetes mellitus, and lack of sexual development |
|  |  | [8] | Dystonia (65.7%), intellectual disabilities (36.8%), sensorineural hearing loss (31.5%), and extrapyramidal rigidity (5.2%) |
|  |  | [9] | Patient 1: Dystonia, gait difficulties, mild cerebral atrophy, and decline in language and cognitive functions  Patient 2: Asymptomatic |
|  |  | [10] | sensorineural hearing loss and dystonia |
|  |  | [11] | Dystonia and abnormalities in pattern reversal visual EPs (PRVEPs), and somatosensory EPs (SSEPs). |
|  |  | [12] | Progressive gait difficulties and truncal dystonia |
| 2 | c.1A>G | [13] | Moderate/mild deafness, hair loss, intellectual disability and failure to develop secondary sexual characteristics |
| 3 | c.270delA | [14] | Ectodermal appendages, deafness, and failure to develop secondary sexual characteristics |
| 4 | c.321 + 1 G > A | [15] | Patient 1: Alopecia, complete edentulism, sensorineural deafness, moderate intellectual disability, primary amenorrhea, and failure to develop secondary sexual characteristics  Patient 2: Alopecia, partial edentulism, sensorineural deafness, moderate intellectual disability, blepharospasm, primary amenorrhea, and failure to develop secondary sexual characteristics  Patient 3 and 4: Alopecia, sensorineural deafness, moderate intellectual disability, and failure to develop secondary sexual characteristics  Patient 5: Alopecia, sensorineural deafness, moderate intellectual disability, dystonia, choreoathetosis, primary amenorrhea, and failure to develop secondary sexual characteristics  Patient 6: Alopecia, sensorineural deafness, moderate intellectual disability, dystonia, choreoathetosis, spastic quadriplegia, dysarthria, dysphagia, and failure to develop secondary sexual characteristics |
| 5 | c.1091+1G>A | [16] | Patient 1: Slowness of movements, dysarthria, dysphagia, mild intellectual disability, alopecia, hypogonadotropic hypogonadism, diabetes mellitus, hypophonic speech and hypokinesia |
|  |  | [17] | Patient 1:  Slowness of movements, dysarthria, dysphagia, mild intellectual disability, alopecia, hypogonadotropic hypogonadism, diabetes mellitus, hypophonic speech, hypokinesia AND iron deposition in the brain |
| 6 | c.270 dup | [18] | Patient 1: Primary amenorrhea, delayed puberty, learning disabilities, mild intellectual disability, hyper-hypogonadotropic hypogonadism, prepubertal internal genitalia, non-immune-insulinopenic diabetes mellitus, secondary hypothyroidism, sensorineural deafness, and dysarthria |
| 7 | c.127-3delTAGinsAA | [19] | Hypothyroidism, frontotemporal alopecia, dystonia, hypogonadism, and intellectual disability |
| 8 | c.1423‐1_1425delGACA | [20] | Alopecia, intellectual disability, hypogonadism, mild sensory neural deafness, facial dysmorphism, delayed speech, language development, and extrapyramidal features |
| 9 | c.1091+2T>C | [21] | Dystonia, mild intellectual disability, hypergonadotrophic hypogonadism, primary amenorrhea, diabetes mellitus, hypothyroidism, bilateral sensorineural hearing loss and alopecia |
| 10 | c.1488_1489delAG | [22] | Alopecia, hypogonadism, hypothyroidism, intellectual disability, anemia and thrombocytopenia |
| 11 | c.1111delA | [23] | Alopecia, diabetes mellitus, intellectual disability, hypothyroidism and hypogonadism |
| 12 | c.906 G>A | [19] | Patient 1: Alopecia, diabetes mellitus, hypogonadism, intellectual disability, deafness, extrapyramidal signs and myocardial infarction  Patient 2: Alopecia, hypogonadism, intellectual disability and myocardial infarction  Patient 3: Alopecia, diabetes mellitus, hypogonadism, intellectual disability, deafness, extrapyramidal signs and amenorrhea |
| 13 | c.1238delA  c.459- 7_499del | [24] | Patient 1,2: Amenorrhea, alopecia, sensorineural hearing loss, diabetes mellitus and intellectual disability  Patient 3: Alopecia, sensorineural hearing loss, diabetes mellitus and intellectual disability |

References

1. Hdiji O, Turki E, Bouzidi N, Bouchhima I, Damak M, Bohlega S, et al. Woodhouse-Sakati Syndrome: Report of the First Tunisian Family with the C2orf37 Gene Mutation. J Mov Disord. 2016;9:120–3.

2. Almeqdadi M, Kemppainen JL, Pichurin PN, Gavrilova RH. Phenotypic Variability of c.436delC DCAF17 Gene Mutation in Woodhouse-Sakati Syndrome. Am J Case Rep. 2018;19:347–53.

3. Nanda A, Pasternack SM, Mahmoudi H, Ishorst N, Grimalt R, Betz RC. Alopecia and hypotrichosis as characteristic findings in Woodhouse-Sakati syndrome: report of a family with mutation in the C2orf37 gene. Pediatr Dermatol. 2014;31:83–7.

4. Ben-Omran T, Ali R, Almureikhi M, Alameer S, Al-Saffar M, Walsh CA, et al. Phenotypic heterogeneity in Woodhouse-Sakati syndrome: two new families with a mutation in the C2orf37 gene. Am J Med Genet A. 2011;155A:2647–53.

5. Sheridan MB, Wohler E, Batista DAS, Applegate C, Hoover-Fong J. The Use of High-Density SNP Array to Map Homozygosity in Consanguineous Families to Efficiently Identify Candidate Genes: Application to Woodhouse-Sakati Syndrome. Case Rep Genet. 2015;2015:169482–169482.

6. Al-Khawaga S, Khalifa A, Hussain K. Woodhouse-Sakati Syndrome: Clinical and Molecular Study on a Qatari Family with C2orf37 Gene Mutation. European Society for Paediatric Endocrinology; 2018.

7. Ali R, Al-Dewik N, Mohammed S, Elfituri M, Agouba S, Musa S, et al. Expanding on the phenotypic spectrum of Woodhouse-Sakati syndrome due to founder pathogenic variant in DCAF17: Report of 58 additional patients from Qatar and literature review. American Journal of Medical Genetics Part A. 2022;188:116–29.

8. Alazami AM, Al-Saif A, Al-Semari A, Bohlega S, Zlitni S, Alzahrani F, et al. Mutations in C2orf37, encoding a nucleolar protein, cause hypogonadism, alopecia, diabetes mellitus, mental retardation, and extrapyramidal syndrome. Am J Hum Genet. 2008;83:684–91.

9. Alharbi MS. Woodhouse-Sakati syndrome (WSS): A case report of 3 Saudi sisters with urogenital anomalies. Saudi Med J. 2021;42:1237–42.

10. Bohlega S, Abusrair AH, Al-Ajlan FS, Alharbi N, Al-Semari A, Bohlega B, et al. Patterns of neurological manifestations in Woodhouse-Sakati Syndrome. Parkinsonism & Related Disorders. 2019;69:99–103.

11. Abusrair A, AlHamoud I, Bohlega S. Multimodal Evoked Potential Profiles in Woodhouse–Sakati Syndrome. Journal of Clinical Neurophysiology. 2020;Publish Ahead of Print.

12. Alderson J, Ghosh PS. Clinical Reasoning: Seven-year-old girl with progressive gait difficulties. Neurology. 2020;94:364.

13. Shah K, Jan A, Ahmad F, Basit S, Ramzan K, Ahmad W. Woodhouse-Sakati syndrome in a family is associated with a homozygous start loss mutation in the DCAF17 gene. Clin Exp Dermatol. 2020;45:159–64.

14. Ali RH, Shah K, Nasir A, Steyaert W, Coucke PJ, Ahmad W. Exome sequencing revealed a novel biallelic deletion in the DCAF17 gene underlying Woodhouse Sakati syndrome. Clin Genet. 2016;90:263–9.

15. Habib R, Basit S, Khan S, Khan MN, Ahmad W. A novel splice site mutation in gene C2orf37 underlying Woodhouse–Sakati syndrome (WSS) in a consanguineous family of Pakistani origin. Gene. 2011;490:26–31.

16. Kurnaz E, Türkyılmaz A, Yaralı O, Demir B, Çayır A. A novel DCAF17 homozygous mutation in a girl with Woodhouse-Sakati syndrome and review of the current literature. Journal of Pediatric Endocrinology and Metabolism. 2019;32:1287–93.

17. Haeri G, Akhoundi FH, Alavi A, Abdi S, Rohani M. Endocrine Abnormalities in a Case of Neurodegeneration with Brain Iron Accumulation. Mov Disord Clin Pract. 2020;7:706–7.

18. Sendur SN, Oguz S, Utine GE, Dagdelen S, Oguz KK, Erbas T, et al. A case of Woodhouse-Sakati syndrome with pituitary iron deposition, cardiac and intestinal anomalies, with a novel mutation in DCAF17. European Journal of Medical Genetics. 2019;62:103687.

19. Alazami AM, Schneider SA, Bonneau D, Pasquier L, Carecchio M, Kojovic M, et al. C2orf37 mutational spectrum in Woodhouse-Sakati syndrome patients. Clin Genet. 2010;78:585–90.

20. Fozia F, Shah K, Nazli R, Khan SA, Ahmad I, Mohammad N, et al. Novel splicing-site mutation in DCAF17 gene causing Woodhouse-Sakati syndrome in a large consanguineous family. J Clin Lab Anal. 2022;36:e24127–e24127.

21. Louro P, Durães J, Oliveira D, Paiva S, Ramos L, Macário MC. Woodhouse–Sakati Syndrome: First report of a Portuguese case. Am J Med Genet. 2019;179:2237–40.

22. Zhou M, Shi N, Zheng J, Chen Y, Wang S, Xiao K, et al. Case Report: A Chinese Family of Woodhouse-Sakati Syndrome With Diabetes Mellitus, With a Novel Biallelic Deletion Mutation of the DCAF17 Gene. Front Endocrinol (Lausanne). 2021;12:770871.

23. Chen G, Zhou L, Chen Q, Wang J, Jiang P, Shen R, et al. Case Report: A Deletion Variant in the DCAF17 Gene Underlying Woodhouse-Sakati Syndrome in a Chinese Consanguineous Family. Front Genet. 2021;12:741323.

24. Abdulla MC, Alazami AM, Alungal J, Koya JM, Musambil M. Novel compound heterozygous frameshift mutations of C2orf37 in a familial Indian case of Woodhouse–Sakati syndrome. J Genet. 2015;94:489–92.
